# Serological study of *Neospora caninum* infection in dogs in central China

**DOI:** 10.1051/parasite/2016025

**Published:** 2016-06-17

**Authors:** Shuai Wang, Zhijun Yao, Nian Zhang, Dong Wang, Jingbo Ma, Shiguo Liu, Bin Zheng, Bin Zhang, Kuo Liu, Haizhu Zhang

**Affiliations:** 1 Department of Human Parasitology, School of Basic Medical Sciences, Xinxiang Medical University Xinxiang Henan 453003 PR China; 2 Xinxiang Assegal Medical Examination Institute Xinxiang Henan 453003 PR China

**Keywords:** *Neospora caninum*, Dog, Seroprevalence, IFAT, Central China

## Abstract

*Neospora caninum* is a protozoan parasite that causes abortion in cattle as well as reproduction problems and neurological disorders in dogs. Dogs are important in the epidemiology of *N. caninum* because they act as definitive hosts, shedding oocysts in the environment. To investigate the seroprevalence of *N. caninum* infection in dogs in central China, 1176 serum samples were collected from domestic dogs in Henan province, central China between March 2015 and February 2016 and tested for IgG antibody against *N. caninum,* using the indirect fluorescent antibody test (IFAT). The overall seroprevalence of *N. caninum* was nearly 15% (172/1176). No significant difference was observed between this seroprevalence according to sex and breed of dogs (*p* > 0.05). The infection rate in rural dogs (18%) was higher (*p* < 0.05) than in urban dogs (11%). The prevalence of *N. caninum* infection in dogs increased (*p* < 0.05) with age. The results of the present study indicate the high prevalence of *N. caninum* antibodies in dogs in Henan province, central China. Sanitary conditions and animal health must be improved to prevent the transmission risk of *N. caninum* by dogs.

## Abstract (fr)

*Neospora caninum* est un protozoaire parasite qui provoque l’avortement chez les bovins ainsi que des problèmes de reproduction et des troubles neurologiques chez les chiens. Les chiens sont importants dans l’épidémiologie de *N. caninum* parce qu’ils agissent comme hôtes définitifs, qui excrètent des oocystes dans l’environnement. Pour étudier la séroprévalence de l’infection par *N. caninum* chez les chiens dans le centre de la Chine, 1176 échantillons de sérum ont été prélevés sur les chiens domestiques dans la province du Henan, en Chine centrale, entre Mars 2015 et Février 2016 et testé pour les anticorps IgG contre *N. caninum* en utilisant le test d’immunofluorescence indirecte (IFAT). La séroprévalence générale contre *N. caninum* était de 15 % (172/1176). Aucune différence significative n’a été observée pour cette séroprévalence en fonction du sexe et de la race des chiens (*p* > 0,05). Le taux d’infection chez les chiens ruraux (18 %) était plus élevé (*p* < 0,05) que chez les chiens urbains (11 %). La prévalence de l’infection par *N. caninum* chez les chiens augmentait (*p* < 0,05) avec l’âge. Les résultats de la présente étude indiquent la prévalence élevée des anticorps contre *N. caninum* chez les chiens dans la province du Henan en Chine centrale. Les conditions sanitaires et la santé animale doivent être améliorées afin d’éviter le risque de transmission de *N. caninum* par les chiens.

## Introduction

*Neospora caninum* is an intracellular protozoan parasite that causes the disease neosporosis. It has a worldwide distribution and is known to cause neuromuscular disorders in dogs and abortion in cattle, the latter leading to substantial economic losses in the dairy industry [15, 17, 24, 33]. Canids such as dogs, dingoes, gray wolves, and coyotes (but not foxes) are its definitive hosts and a variety of animals ranging from birds to mammals can act as its intermediate hosts [11, 14, 20, 27].

Dogs are important in the epidemiology of *N. caninum* because they act as definitive hosts, shedding oocysts into the environment [20, 23], which is a major risk factor for the occurrence of miscarriages and stillbirths associated with *N. caninum* in cattle and other intermediate hosts [2, 3, 9]. Moreover, the parasite can be transmitted transplacentally in dogs for several generations [6, 7]. Neosporosis can cause severe neuromuscular disorders such as ascending paralysis with hyperextension of the hind limbs, especially in congenitally infected dogs [17].

Antibodies to *N. caninum* have been reported in dogs worldwide [21, 28, 29]. There have also been some surveys of *N. caninum* infections in dogs in some provinces or cities of China in recent years (Table 1, [1, 16, 18, 19, 26, 30, 32, 35]). However, little information is available on the epidemiology of *N. caninum* infections in dogs in central China. In 2014, Yang et al reported the infection rate of *N. caninum* in dogs from Henan province to be 0.0% (0/31) [35]. But only 31 serum samples of dogs from Henan province were tested for antibodies to *N. caninum* in Yang et al.’s study [35]. The number of serum samples tested was too small to objectively reflect the overall infection status of *N. caninum* in dogs in Henan province. Therefore, the objective of the present survey was to determine the seroprevalence of *N. caninum* in domestic dogs in Henan province, central China.

**Table 1.** The prevalence of *Neospora caninum* infection in dogs in the People’s Republic of China.

Provinces/cities	Year of sampling^1^	No. tested	Positive (%)	Method^2^	Reference
Qinghai	2006	80	25 (31.25)	ELISA	[26]
Urumchi	<2010	29	5 (17.24)	ELISA	[1]
Shenyang	2012	212	74 (34.91)	Nested-PCR	[18]
Henan	2013	31	0 (0.0)	NAT	[35]
Anhui	2013	22	0 (0.0)	NAT	[35]
Jilin	2013	43	6 (13.95)	NAT	[35]
Beijing	2013	186	1 (0.54)	ELISA	[16]
Yanbian	<2015	27	3 (11.11)	ELISA	[30]
Kuerle	2015	61	14 (22.95)	ELISA	[32]
Chuzhou	2013	89	3 (3.37)	Nested-PCR	[19]
Bengbu	2013	31	2 (6.45)	Nested-PCR	[19]

1 Years of sampling are listed as published in the references. In cases where this information was not available, the year listed here is the year when the study was published, as indicated by “<”.

2 ELISA: enzyme-linked immunosorbent assay; PCR: polymerase chain reaction; NAT: *Neospora* agglutination test.

## Materials and methods

### Ethics statement

The study was reviewed and approved by the Ethics Review Committee of the Xinxiang Medical University (Reference No. 2015018).

### The study site

The study was conducted in Henan province, located in the central part of mainland China, covering an area of 167,000 km^2^, with a population of approximately 106.01 million. Its geographical position is at east longitude 110°21′–116°39′ and at north latitude 31°23′–36°22′. The Yellow River passes through central Henan. The area has a continental monsoon climate, with four distinctive seasons. There are 17 provincial cities distributed across Henan province, with the city of Zhengzhou as its capital. Five cities including Anyang (35°13′–36°22′ N, 113°37′–114°58′ E), Sanmenxia (33°31′–35°05′ N, 110°21′–112°01′ E), Zhengzhou (34°16′–34°58′ N, 112°42′–114°13′ E), Xinyang (31°46′–31°52′ N, 114°01′–114°06′ E), and Shangqiu (33°43′–34°52′ N, 114°49′–116°39′ E), located in the northern, western, central, southern, and eastern parts of Henan province, were selected for sample collections.

### Sample collection

A total of 1176 blood samples from domestic dogs were collected in these five cities in Henan province between March 2015 and February 2016. Dog owners were asked for details on the animal’s age, sex, source, breed, rearing conditions, and medical history, using a structured questionnaire. Blood samples were centrifuged and sera were recovered and transferred to 1.5 mL Eppendorf tubes. All sera were then stored at −80 °C until testing for the anti-*N*. *caninum* antibodies.

### Determination of antibodies against *N. caninum*

An indirect fluorescent antibody test (IFAT) was performed to detect anti-*N*. *caninum* antibodies in dog sera. The test procedures were performed using a *Neospora caninum* FA Substrate Slide according to the manufacturer’s instructions (Catalog No. SLD-IFA-NC, VMRD Inc, Washington, USA). Teflon-masked slides were coupled with *N*. *caninum* NC-1 tachyzoites maintained in Vero cells. Anti-Canine IgG FITC Conjugate (Catalog No. 035-10, VMRD Inc, Washington, USA) was used as secondary antibody. On each slide, sera from canines positive for *N*. *caninum* and from canines negative for *N*. *caninum* (Catalog No. 211-P-NC-CAN and 211-N-NC-CAN, VMRD Inc, Washington, USA) were included as a control. Dog sera were screened at a titer of 1:50 cut-off value [28, 34].

### Statistical analysis

Differences in *N. caninum* prevalence for certain variables such as age, breed, and sex were analyzed using a chi square test. Statistical analysis was performed using SPSS 20 software for Windows (SPSS Inc, Chicago, Illinois, USA). The differences were considered statistically significant if *p* < 0.05.

## Results

In the present study, 1176 dogs were tested for the presence of antibodies against *N. caninum* using the IFAT. As shown in Table 2, the overall recorded seroprevalence of *N. caninum* in dogs in Henan province, central China was 14.63% (172/1176). Seropositive dogs from different cities were: 16.07% of 224 from Anyang, 20.43% of 235 from Sanmenxia, 14.84% of 256 from Zhengzhou, 9.50% of 242 from Xinyang, and 12.33% of 219 from Shangqiu.

**Table 2.** Seroprevalence of *Neospora caninum* infection in dogs in Henan province, central China.

Variable	No. examined	No. positive	Prevalence (%)	*X*^2^	*p*-value
Region					
Anyang	224	36	16.07	12.724	0.013
Sanmenxia	235	48	20.43		
Zhengzhou	256	38	14.84		
Xinyang	242	23	9.5		
Shangqiu	219	27	12.33		
Sex					
Male	617	97	15.72	1.247	0.264
Female	559	75	13.42		
Breed					
Pure breed	742	101	13.61	1.656	0.198
Cross-breed	434	71	16.36		
Area					
Urban	609	69	11.33	10.988	0.001
Rural	567	103	18.17		
Age (years)					
≤3	318	32	10.06	9.043	0.011
3 ~ 6	573	87	15.18		
≥6	285	53	18.6		
Total	1176	172	14.63		

The seroprevalence of *N. caninum* in males was 15.72% (97/617) and in females 13.42% (75/559) (Table 2). Although the seroprevalence in males was higher than the females, the difference was not significant (*p* > 0.05).

The seroprevalence of *N. caninum* infection was 13.61% (101/742) in purebred dogs and 16.36% (71/434) in cross-breed dogs, showing no significant difference by breed (*p* > 0.05). The prevalence of *N. caninum* infection in rural dogs (18.17%) was significantly higher compared to that of dogs raised in urban areas (11.33%) (*p* < 0.01).

The prevalence of *N. caninum* infection in dogs increased significantly (*p* < 0.05) with increasing age. The highest prevalence of infection (18.60%) was detected in six-year-old or older dogs, followed by intermediate prevalence (15.18%) in the 3–6 year age group, while the prevalence found in dogs in the ≤3 year age group was 10.06% (Table 2).

## Discussion

The detection of antibodies against *N. caninum* has been undertaken worldwide in dogs [25, 28, 29, 34]. The reference method for this detection is the IFAT [5, 8, 28]. A 1:50 cut-off in IFAT has been recommended and commonly used for canine sera [25, 28, 34]. Hence, in the present study, we used the IFAT to determine the seroprevalence of *N. caninum* in dogs and used a titer of 1:50 as the positive threshold titer.

Out of the 1176 dog serum samples tested in this study, 172 were classified as seropositive for *N. caninum*, giving a seroprevalence of 14.63%. The prevalence of 14.63% was lower than the prevalence of 21.7% in central Poland [12], 32.0% in Italy [28], and 19.4%–33.0% in Iran [10, 12, 13, 22], but higher than the reported prevalence of 3.6% in Korea [25], 7.9% in Portugal [21], and 12.4% in Brazil [31]. These differences, as previously commented, may be explained by the use of different serological tests, survey periods, sample sizes, and type of dog population.

In the present study, a higher prevalence rate of *N. caninum* was found in males than in females, but the difference was not significant (*p* > 0.05). These findings are consistent with the observations made by others [21, 25]. However, some researchers found female dogs to be affected more than males [12, 31].

Most of the reports find no specific breed susceptibility or a higher seroprevalence of *N. caninum* in mixed breed dogs [21, 23]. In the present study, although the seroprevalence in cross-breed dogs was higher than in purebred dogs, the difference was not significant (*p* > 0.05). However, Robbe et al. have suggested higher seropositivity in purebred dogs [28]. The role of breeds in the epidemiology of canine neosporosis is not well established and requires further research.

The present survey also showed that *N. caninum* seroprevalence was higher in rural dogs than in urban dogs (*p* < 0.01). This interesting finding may be attributed to feeding habits (like eating raw meat containing parasite cysts), differences in welfare, and different living environments.

In the present study, there appears to be a strong tendency for elevated risk of pathogen contact with increasing age, suggesting postnatal exposure to *N. caninum*. Similar observations from Brazil [4], Poland [12], and Iran [10] confirm this finding.

In conclusion, a high prevalence of *N. caninum* infection was found in domestic dogs in Henan, central China. Sanitary conditions and animal health must be improved to prevent the transmission risk of *N. caninum* by dogs.

## Conflict of interest

The authors declare no conflict of interest in relation with this paper.
